# Herbal medicine for the management of polycystic ovary syndrome (PCOS) and associated oligo/amenorrhoea and hyperandrogenism; a review of the laboratory evidence for effects with corroborative clinical findings

**DOI:** 10.1186/1472-6882-14-511

**Published:** 2014-12-18

**Authors:** Susan Arentz, Jason Anthony Abbott, Caroline Anne Smith, Alan Bensoussan

**Affiliations:** National Institute of Complementary Medicine, University of Western, Locked Bag 1797, Penrith South, Sydney, NSW 2751 Australia; School of Women’s and Children’s Health, University of New South Wales, Sydney, Sydney, Australia; National Institute of Complementary Medicine (NICM), University of Western Sydney, Sydney, Australia

## Abstract

**Background:**

Polycystic ovary syndrome (PCOS) is a prevalent, complex endocrine disorder characterised by polycystic ovaries, chronic anovulation and hyperandrogenism leading to symptoms of irregular menstrual cycles, hirsutism, acne and infertility. Evidence based medical management emphasises a multidisciplinary approach for PCOS, as conventional pharmaceutical treatment addresses single symptoms, may be contra-indicated, is often associated with side effects and not effective in some cases. In addition women with PCOS have expressed a strong desire for alternative treatments. This review examines the reproductive endocrine effects in PCOS for an alternative treatment, herbal medicine. The aim of this review was to identify consistent evidence from both pre-clinical and clinical research, to add to the evidence base for herbal medicine in PCOS (and associated oligo/amenorrhoea and hyperandrogenism) and to inform herbal selection in the provision clinical care for these common conditions.

**Methods:**

We undertook two searches of the scientific literature. The first search sought pre-clinical studies which explained the reproductive endocrine effects of whole herbal extracts in oligo/amenorrhoea, hyperandrogenism and PCOS. Herbal medicines from the first search informed key words for the second search. The second search sought clinical studies, which corroborated laboratory findings. Subjects included women with PCOS, menstrual irregularities and hyperandrogenism.

**Results:**

A total of 33 studies were included in this review. Eighteen pre-clinical studies reported mechanisms of effect and fifteen clinical studies corroborated pre-clinical findings, including eight randomised controlled trials, and 762 women with menstrual irregularities, hyperandrogenism and/or PCOS. Interventions included herbal extracts of *Vitex agnus-castus*, *Cimicifuga racemosa*, *Tribulus terrestris*, *Glycyrrhiza spp*., *Paeonia lactiflora* and *Cinnamomum cassia*. Endocrine outcomes included reduced luteinising hormone (LH), prolactin, fasting insulin and testosterone. There was evidence for the regulation of ovulation, improved metabolic hormone profile and improved fertility outcomes in PCOS. There was evidence for an equivalent effect of two herbal medicines and the pharmaceutical agents bromocriptine (and *Vitex agnus-castus*) and clomiphene citrate (and *Cimicifuga racemosa*). There was less robust evidence for the complementary combination of spirinolactone and G*lycyrrhiza spp.* for hyperandrogenism.

**Conclusions:**

Preclinical and clinical studies provide evidence that six herbal medicines may have beneficial effects for women with oligo/amenorrhea, hyperandrogenism and PCOS. However the quantity of pre-clinical data was limited, and the quality of clinical evidence was variable. Further pre-clinical studies are needed to explain the effects of herbal medicines not included in this review with current clinical evidence but an absence of pre-clinical data.

**Electronic supplementary material:**

The online version of this article (doi:10.1186/1472-6882-14-511) contains supplementary material, which is available to authorized users.

## Background

Polycystic ovary syndrome (PCOS) is a complex, common reproductive and endocrine disorder affecting up to 17.8% of reproductive aged women [1]. Medical management places strong emphasis on a multidisciplinary approach as pharmaceutical treatments appear to be only moderately effective in treating individual symptoms [2, 3]. Conventional pharmaceutical management is limited by the prevalence of contraindications in women with PCOS [3], non-effectiveness in some circumstances [4], side effects [5] and by preferences of women with PCOS for alternatives to pharmaceutical management [6]. This review examines the mechanisms of effect for a potential alternative treatment, herbal medicine, and reveals six herbal medicines with both pre-clinical and clinical data explaining the reproductive endocrinological effects in PCOS and associated oligo/amenorrhoea and hyperandrogenism.

Complementary medicine (CM) use by women has increased during the past ten years [7–11] with rates of use ranging between 26% and 91% [8, 9]. One of the popular types of CM is herbal medicine [11, 12]. Herbal medicines are known to contain pharmacologically active constituents with physiological effects on female endocrinology and have been positively associated with reduced incidences of breast cancer, osteoporosis and cardiovascular disease [13–18].

PCOS is a life-long condition and although the exact cause is yet to be identified, it is believed to have epigenetic origins, influenced by the uterine environment and behavioural factors [19]. Being overweight exacerbates all aspects of PCOS due to underlying metabolic disturbances [3]. Signs and symptoms are mediated by hormonal disorder including elevated androgens and fasting insulin, and abnormal relative ratio of the gonadotropins luteinising hormone (LH) and follicle stimulating hormone (FSH) [19]. Endocrine imbalances occur within the framework of disordered ovarian folliculogenesis, chronic anovulation, clinical signs of hyperandrogenism and metabolic syndrome [19].

Pharmaceutical treatment for menstrual irregularity includes the oral contraceptive pill (OCP) and ovulation induction with clomiphene citrate (clomiphene) [20, 21] depending on fertility needs. Women with PCOS are however likely to exhibit contraindications for the OCP [3] and whilst induction of ovulation with clomiphene has demonstrated success, pregnancy rates remain inexplicably low [4]. Up to thirty 30% of women, particularly overweight women with PCOS, fail to respond to clomiphene therapy [4, 22, 23]. Management for hyperandrogenism includes anti-androgens and hypoglycaemic pharmaceuticals such as metformin [24]. Metfomin has demonstrated effectiveness for improving insulin sensitivity and hyperandrogenism, however use of metformin is associated with the high incidence of adverse effects including nausea, vomiting and gastro-intestinal disturbances [5].

Herbal medicines are complex interventions with the potential for synergistic and antagonistic interactions between compounds [25]. Effects within the body may also exhibit complexity by simultaneous interactions with various body systems, both biochemically and by altering organ function [26]. The focus of this review was studies investigating whole herbal medicine extracts with direct effects on reproductive endocrinology for the treatment of women with irregular menstruation, hyperandrogenism and PCOS. The rationale for using this methodology was to identify herbal medicines with current scientific evidence explaining specific reproductive endocrinological effects in PCOS, oligo/amenorrhoea and hyperandrogenism, to develop understanding for the direct effects of herbal medicines on reproductive endocrinology and to highlight herbal medicines for which there was current scientific evidence supporting herbal medicine selection. The purpose of this review is to inform clinical decisions in integrative settings and meet clinicians and consumers preferences for pragmatic herbal management within an holistic, individualised treatment frame [27, 28].

We compared laboratory evidence including scientific studies using cell culture and animal models, with clinical data for proof-of-concept effects. A narrative synthesis of pre-clinical studies explaining reproductive endocrinological effects for herbal medicines with corroborative clinical evidence is presented.

## Methods

We used the following definitions. PCOS according to the Rotterdam diagnostic criteria, specified by the presence of two out of three features; oligo/amenorrhoea, hyperandrogenism and polycystic ovaries on ultrasound [29, 30]. Associated endocrine features for PCOS included elevated LH [31], which is strongly associated with infertility (p = 0.0003) [32] and miscarriage [33] and elevated fasting glucose which is prevalent in approximately 31% of women with PCOS including normal weight women [34].

Oligomenorrhoea was defined as menstrual cycle length that extended beyond 35 days (day one being the first day of menses). Amenorrhoea was defined as no menstrual period for three to six months or more [19]. This review was focussed on hypothalamic, pituitary and ovarian causes of menstrual irregularity with associated elevated gonadotropins including LH and prolactin and arrested folliculogenesis typically observed in polycystic ovaries. Hyperprolactinaemia is usually considered a unique cause for oligo/amenorrhoea; however in the present case it was included due to the potential co-existence for elevated prolactin, LH and PCOS, [32, 35].

Hyperandrogenism was defined as clinical or biochemically excessive androgens. Clinical markers in females include cutaneous manifestations such as the presence of acne, hirsutism and/or male pattern alopecia. Biochemical indications include elevated plasma concentration of androgens.

We conducted two searches. The first was sensitive and aimed to capture all pre-clinical studies explaining the reproductive endocrine effects of whole herbal extracts in PCOS or associated oligo/amenorrhoea and hyperandrogenism. The second search was specific and sought only clinical studies investigating herbal medicines revealed during the first search (for which a mechanism of effect had been demonstrated). We additionally searched, on a case by case basis for pre-clinical evidence for herbal medicines identified during the second search, but not included in the results of the first search. Clinical studies were excluded based on the absence of evidence for a mechanism of effect for the whole herbal extract in reproductive endocrinology associated with PCOS, oligo/amenorrhoea and hyperandrogenism. We used this approach to improve transparency and to limit confirmation bias for herbal medicines favoured by the authors in clinical practice.

The first search revealed ten herbal medicines with a demonstrated mechanism of reproductive endocrinological effect for the whole herbal extract in PCOS, oligo/amenorrhoea and hyperandrogenism. These were *Cimicifuga racemosa*, *Cinnamomum cassia, Curcuma longa*, *Glycyrrhiza spp*., *Matricaria chamomilla*, *Mentha piperita*, *Paeonia lactiflora, Silybum marianum, Tribulus terrestris and Vitex agnus-castus*. Herbal medicines with a demonstrated mechanism of effect were entered as key terms in the second search.

We searched the following electronic databases: the Cochrane Library, MEDLINE ovidSP, CINAHL (1936 to present), SciVerse, EMBASE, PubMed, from the date of database inception to June 2014. In addition, we manually searched bibliographies of review articles.

Key terms for the first search included: title or abstract CONTAINS ‘herbal medicine’ OR ‘herbal extract*’ OR ‘phytotherapy’ OR ‘botanical’ AND title or abstract CONTAINS ‘androgen*’ OR ‘oestrogen*’OR ‘follicle stimulating hormone’ OR ‘luteinising hormone’ OR ‘prolactin’ OR ‘insulin’ OR ‘glucose’ OR ‘polycystic ‘ovar*’. Search terms for the second search included the following key words in the title or abstract, CONTAINS; ‘menstrual irregularity’ OR ‘oligomenorrhoea’ OR ‘amenorrhoea’ OR ‘hyperandrogenism’ OR ‘hirsutism’ OR ‘acne’, OR ‘polycystic ovary syndrome’ OR ‘PCOS’ OR ‘polycystic ovar*’ OR ‘oligo-ovulation’ OR ‘anovulation’ OR ‘fertility’ OR ‘infertility’ OR ‘pregnancy’ AND ten herbal medicines identified from the laboratory search; ‘*Cimicifuga racemosa’* OR ‘*Cinnamomum cassia’* OR *‘Curcuma longa’* OR ‘*Glycyrrhiza ‘* OR *Matricaria chamomilla* OR *‘Mentha piperita’* OR ‘*Paeonia lactiflora’* OR *‘Silybum marianum’* OR ‘*Tribulus terrestris’* OR ‘*Vitex agnus-castus’*. Truncation was used to capture plural key words and synonyms, and acronyms were used for some hormones (FSH and LH).

Our laboratory search included investigations into the effects of herbal medicine using computer models, cell cultures, animals with PCOS induced with oestradiol valerate and androgens and sterilised and ovariectomised rats. We excluded laboratory studies which commenced using isolated chemicals not directly extracted from crude herbal medicines and studies examining androgen effects in male animals.

Our second search for clinical trials was performed without language restriction and included randomised controlled trials, non-randomised, open label and single arm clinical trials. We included clinical studies investigating commercially available herbal extracts and investigations that compared the effectiveness of herbal medicine with pharmaceuticals. We excluded clinical studies investigating herbal medicines with unrelated outcomes (including pre-menstrual syndrome, endometriosis and mastalgia) and clinical studies examining the effectiveness of complex herbal formulas for PCOS and associated oligo/amenorrhoea and hyperandrogenism, without demonstration of a mechanism of effect for the whole complex formula. We compared data from laboratory and animal studies with the outcomes of clinical trials. Clinical studies were assessed for risks of bias at study and outcome levels with risks summarised, tabulated (Tables 1 and 2) and presented in contextual narrative.

**Table 1:** **Summary of evidence for the reproductive endocrinological effects of six herbal medicines in oligo/amenorrhoea, hyperandrogenism and PCOS**

Herbal medicine	Evidence		Physiological effects in menstrual irregularity (oligo/amenorrhoea), hyperandrogenism and/or PCOS.
Botanic name			
	*Pre-clinical in vitro and in vivo*	*Clinical RCTs (detailed in Table* 2 *)*	
Herbal extract			
*Vitex agnus-castus*	Eight studies investigated gonadotropic hormone concentration effects of *Vitex agnus-castus*.	Three RCTs investigate clinical effectiveness for *Vitex agnus-castus* for oligo/amenorrhoea and PCOS [61, 62, 64]. One RCT demonstrated equivalence for Bromocriptine and *Vitex agnus-castus*[63].	
Ethanol extracts			
Strontan®, Mastodynon®, Phyto-hypophyson®, Agnacaston®	1. Investigation for equivalence of dopaminergic effects for *Vitex agnus-castus* and the pharmaceutical Lisuride using rat pituitary cell cultures (basal and stimulated cells) [41]		1. Lowers prolactin due to dopaminergic effects [38–41, 63]
	2. Brain (calf, guinea pig and rats) receptor tissue cultures including DA2, histamine and 5HT transporters. Radio ligand and super-fusion experiments [40]		2. No change for serum prolactin [64]
	3. Three investigations found affinity for *Vitex agnus-castus* and β oestrogen receptors [38, 43, 69]		3. FSH no change [39]
	4. Using recombinant human dopamine (DA2) receptor proteins [38]		4. LH no change [39]
	5. The affinity of *Vitex agnus-castus* extract (with and without fatty acids) for human μ opoid receptor cells cloned and transfected into hamster ovary cells [70]		5. LH lowered [49]
	6. The endocrine effects for *Vitex agnus-castus* were investigated in normal and ovariectomised rats [49]		6. Binds to β oestrogen receptors [38, 43, 69]
	7. Corpus striatum membrane including D2 receptors to assess the inhibitory properties of *Vitex agnus-castus* on prolactin, FSH and LH [39]		7. Increased serum oestradiol [49, 64]
			8. Increased serum progesterone [49, 62]
			9. Improved pregnancy rates [61, 62]
*Cimicifuga racemosa*	Four laboratory studies investigated pituitary oestrogen receptor binding and gonadotropin concentrations following exposure to *Cimicifuga racemosa.*	Three RCTs demonstrate positive fertility effects for *Cimicifuga racemosa* in women with PCOS [65, 67, 68]	
Ethanol extract			
Klimadynon®			
	1. One study investigated a constituent flavonoid of *Cimicifuga racemosa*, discovered during the course of the study for reduction for LH in ovariectomised rats [37]		1. Binds with α oestrogen receptors [44] in the pituitary and reduces LH secretion [45, 52, 68].
	2. Oestrogen receptor binding affinity for *Cimicifuga racemosa* was studied using pituitary cell cultures from ovariectomised rats. This study followed a clinical study demonstrating significantly lowered LH in post-menopausal women following administration of *Cimicifuga racemosa* (2 mg for two months) against placebo control (n = 110) [45]		2. Increases luteal progesterone concentration [65, 67, 68]
	3. Binding affinity for oestrogen receptors (ERα) for *Cimicifuga racemosa* examined using MCF7 cell cultures [44]		3. Improves endometrial thickness for infertile women with PCOS [65, 67, 68].
	4. Chronic and acute dosage effects of *Cimicifuga racemosa* and oestradiol on oestrogen receptors, gene expression, uterine and bone tissue of ovariectomised rats [52]		4. Lowers LH in women with PCOS [65, 67, 68]
			5. Improves FSH:LH ratio for women with PCOS [67]
			6. Limits anti-oestrogen effects when used in combination with Clomiphene citrate for women with PCOS [65, 68]
*Cinnamon cassia*	One animal study compared the effectiveness of *Cinnamomum cassia* with metformin against controls in rats with PCOS. Hormone concentration was measured at 15 and 30 days [48]	One pilot RCT demonstrated positive effects for metabolic parameter’s (HOMO and QUICKI) for *Cinnamomum cassia* in overweight women with PCOS [66]	1. Equivalence for metformin for reduced testosterone in PCOS [48]
*Aqueous extract (animal study)*			
			2. Equivalence for metformin for reduced LH in PCOS [48]
*Ethanol extraction (Human trial)*			
			3. Equivalence for metformin for reduced LH in PCOS [48]
			4 Equivalence for metformin for reduced insulin resistance [48]
			5. Improved metabolic profile for overweight women with PCOS [66]
**Herbal medicine**	**Evidence**		**Physiological effects in oligo/amenorrhoea, hyperandrogenism and/or PCOS**
	***Pre-clinical in vitro and in vivo***	**Data from clinical studies (non RCTs)**	
**Botanic name**			
**Herbal extract**			
*Tribulus terrestris*	Three animal studies investigated the effects of *Tribulus terrestris*, two for polycystic ovaries and one on oestrogen sensitive tissues in rats.	Two clinical studies	
Ethanol extracts	1. One study examined the oestrogenic effects of *Tribulus terrestris* on uterine and vaginal tissue of ovariectomised rats [51].	1. Healthy women n = 8 early menstrual cycle (follicular phase) Pre and post serum hormone concentration for FSH, LH testosterone and oestradiol at 8 am and 12 pm. Intervention consisted of *Tribulus Terrestris* 250 mg per day over five days. Results showed significant increase in FSH and rise in LH (not significant), an increase in oestradiol and no change in testosterone concentration [56]	1. Ovulation induction in polycystic ovaries [46, 47].
	2. Two studies investigated the ovulation rates, number of corpus luteum and follicle characteristics in rats with polycystic ovaries following exposure to various doses of *Tribulus terrestris*[46, 47].	2. Equivalence of *Tribulus terrestris* and three ovulation induction pharmaceuticals evaluated ovulation in women with oligo/anovular infertility (n = 148) [60].G	2. No oestrogenic effects in female reproductive tissues [51].
			3. Increased FSH in healthy women [56].
			4. Equivalence for ovulation induction for *Tribulus Terrestris* and Clomiphene for women with oligo/anovular infertility [60].
*Glycyrrhiza glabra (European liquorice)*	Two preclinical studies investigated the effects of Glycyrrhiza spp. for steroid hormone concentration and in polycystic ovaries.	Two clinical trials	
	1. Steroid hormone concentration in sterilised and oophrectomised rats following exposure to *Glycyrrhiza spp.* (kanzo) [53].	1. Single arm clinical trial investigating serum androgen concentration in healthy women aged 22–26, (n = 9) following administration of *Glycyrrhiza spp.* 7grams per day [55].	1. Increased aromatisation of testosterone to 17 beta oestradiol shown by significantly dose dependent reduced testosterone and increased oestradiol [53].
*Glycyrrhiza uralensis (Chinese liquorice)*			
Ethanol extract			
Aqueous extract used in two pre-clinical studies			
	2. Morphological features of polycystic ovaries of rats following exposure to two Chinese herbal compounds with only *Glycyrrhiza spp.* as a common ingredient [50].	2. Single arm clinical trial including women with PCOS (n = 32) taking Spirinolactone [54].	2. Reduced free and total testosterone [53].
			3. Reduced serum androgens in healthy women [55].
			4. Reduced androgen flare for women with PCOS using the anti-androgen pharmaceutical Spirinolactone [54].
			5. Improved ovulation rates in polycystic ovaries [50].
*Paeonia lactiflora* in combination with *Glycyrrhiza spp.* Aqueous extract Shakuyaku- kanzo-to (TJ-68)	One laboratory study examined the effects for the combination *Paeonia lactiflora* and *Glycyrrhiza uralensis* on testosterone, oestradiol, FSH and LH in sterilised female rats [53].	Two single arm clinical trials examined androgen concentrations Following treatment with *Paeonia lactiflora* and *Glycyrrhiza uralensis* in the Chinese herbal combination Shakuyaku-kanzo-to. One included infertile oligomenorrhoeic women with hyperandrogenism (n = 8) [58] and the other included women with oligo/amenorrhoea and PCOS (n = 34) [59].	1. Reduced total and free testosterone [53, 58, 59].
			2. Increaed SHBG [59].
			3. Reduced LH [53].
			4. Reduced LH:FSH ratio [59].
			5. Oestradiol slight increase (not significant) [53].
			6. Improved ovulation in women with PCOS [58].
*Paeonia lactiflora* in combination with *Cinnamomum cassia* Aqueous extract Unkei-to	*Paeonia lactiflora* and *Cinnamomum cassia* combination was investigated for steroid hormonal effects on cultured human granulosa cells (obtained from women undergoing IVF). Cells were incubated with different doses for 48 hours [42]	One clinical trial investigated the effects of *Paeonia lactiflora* and *Cinnamomum cassia* combination (Unkei-to) [57]. This single arm study included amenorrheic women aged 17–29 years (n = 157) with a sub group of women with hyper-functioning oligo/amenorrhoea (n = 42). Ovulation occurred in 61.3% of primary amenorrheic women and in 27.3% of secondary amenorrheic women following two months of treatment [57].	1. Increased granulosa production of oestradiol [42].
			2. Increased granulosa production of progesterone [42].
			3. Reduced LH in oligo/amenorrhoea [57].
			4. Improved ovulation rates in oligo/amenorrhoea [57].

**Table 2:** **Summary of randomised controlled trials for five herbal medicines in oligo/amenorrhoea, hyperandrogenism and PCOS**

Author and year of publication	Study design and duration	Subjects	Intervention	Outcome measures	Results and level of significance	Comments
Kilicdag [63]	Randomised comparative effectiveness trial.	Eighty women, 40 with hyperprolactin-aemia, 40 with cyclical mastalgia.	Herbal extract *Vitex agnus-castus* 40 mg in the commercial preparation Agnucaston® by Biomeks, Germany.	Comparison of difference between *Vitex agnus-castus* and Bromocriptine for serum prolactin concentration on days 5–8 of the menstrual cycle.	Mean prolactin concentration before and after in the *V.agnus-castus* arm; 946mIU/L (±173.5) to 529mIU/l (±279.7), p < 0.0001. In the Bromocriptine arm; 885.0 mIU/l (±177.5) to 472.68mIU/L (±265.6), p < 0.0001.	All participants completed the trial. Adverse reactions; zero reported in *V. agnus-castus* group; 12.5% of participants reported adverse reactions in the Bromocriptine group (nausea and vomiting).
	Treatment for 3 months.		1 tablet per day. Bromocriptine in the form of Parlodel produced by Novartis, Turkey, 2.5 mg twice daily.	Normal range 25.2mIU/l - 628.5 mIU/l.	Equivalence demonstrated for the significant reduction of serum prolactin for *V. agnus-castus* and Bromocriptine (P = 0.96).	Small sample sizes with 2 sub-groups. Insufficiently powered to correctly identify the effects; 377 participants were required (±5%, 95% confidence).
Gerhard I, Patek A, et al. [61]	Randomised, double blind, placebo controlled trial.	Ninety-six women with fertility disorders and confirmed infertility (2 years).	*Vitex agnus-castus* 32.4 mg/d in the commercial preparation Mastodynon® liquid extract produced by Bionorica, Germany.	Spontaneous menstruation, luteal phase length, serum hormone concentrations and pregnancy rates.	Non-significant improvement in clinical parameters in 57.6% of women in treatment group versus 36.0% in placebo group, P = 0.069.	Numbers too small for statistical significance in clinical outcomes.
	Three months. Follow up at 2 years	Secondary amenorrhoea, n = 38; luteal insufficiency, n = 31; idiopathic infertility, n = 27.	30 drops per day over 3 months.	Hormonal data from 32 cases. In the third treatment month 66 complete data sets were available.	In a subgroup of women with luteal insufficiency (n = 21) there were significant improvements in clinical parameters in the treatment group compared to placebo (p = 0.023).	Preparation ‘Mastodynon’ contains V agnus-castus plus other herbal extracts which may have confounded outcome measures.
			Mastodynon® additionally contains herbal extracts of *Caulophyllum thalictroides*, *Lilium majus*, Cyclamen, Ignatia and Iris.	Reasons were as follows; 4 due to drug reactions and 15 due to pregnancy.	15 women conceived in the treatment group compared to 8 in placebo group in the first 3 months (while women were treated).	Inconsistencies in data assessment include the recommendation for treatment with Mastodynon over 3–6 months yet it was tested for 3 months.
			No evidence that therapeutic agents additional to *V. agnus-castus* in Mastodynon® affect prolactin concentration.	Four withdrew for unknown reasons.	All pregnant women were withdrawn from the study. 4 women had miscarriages, all in the active arm. After 2 years there were 21 more pregnancies with 2 miscarriages – evenly spread over active and placebo groups.	Women with infertility were included in this study however data from women who conceived were excluded. This may have led to an underestimation of treatment effect (type 1 error).
Bergmann J, Luft B, et al. [62]	Randomised, placebo controlled double blind study. Three months or 3 menstrual cycles.	Women with fertility disorders, (n = 67). Two sub-groups.	Herbal extract Phyto-Hypophyson® by Steril-Pharma GmbH Herrsching, Germany; contains *Vitex agnus-castus* plus *Chelledonium majus* and *Silybum marianum* (St Mary’s thistle) in homeopathic form. Additional herbal extracts have reported activity in hepatic function. There are no reports for direct reproductive effects. 150 drops per day (7.5 ml per day).	Primary outcome for participants with amenorrhoea: at least one spontaneous menses.	Oligomenorrhoeic subgroup - clinical outcomes were significantly improved in the treatment arm at 82% compared to 45% in placebo arm P = 0.021. When the amenorrheic group were included in analysis, differences were not significant p = 0.19.	Diagnosis for anovulatory amenorrhoea is not well described. Non-statistically significant take home baby rates were complicated by insufficient sample size. 366 patients are required to have a 95% chance, as significant at the 5% level, an increase in take home baby rates from 6% in the placebo group to 18% in the experimental group. The authors conclude that this preparation may be useful if given 3–6 months, yet they only tested for 3 months.
		1.oligomenorrhoea, n = 37		For progesterone <1 ng/mL: an increase to >5 ng/mL at the end of 3rd cycle	Mid luteal progesterone concentration in oligomenorrhoeic sub-group was significantly higher than the placebo group p = 0.0479	
		2. amenorrhoea n = 30. Oligomenorrhoea group: Treatment n = 17. Placebo n = 20. Amenorrhoea group. Treatment n = 16. Placebo n = 14.		For oligomenorrhoea: Shortened menstrual cycle of at least 4 days. Earlier ovulation of at least 3 days. For anovulatory oligomenorrhoea: Mid luteal progesterone increase (>50% 5–10 days before menstruation. Secondary clinical outcomes, pregnancy rates and take home baby rates.	At 6 months following conclusion of treatment, the take home baby rate with treatment was 18.7% compared to 6.4% in placebo group. Not statistically significant.	
Milewicz A, Gejdel E, et al. [64]	Randomised placebo controlled, double blind, trial. Three months.	52 women with latent hyperprolactinaemia and luteal phase defects. Participants stratified for cycle length, height (cm) and weight (kgs) and randomised. Baseline differences between arms were not significant p = 0.63, p = 0.48 and p =0.37 respectively. 37 complete case reports: Treatment arm n = 17, placebo n = 20.	*Vitex agnus-castus* extract 20 mg in the commercial preparation of Strotan® Hersteller: Pharma Stroschein GmbH, Hamburg, Germany. 1 capsule per day or placebo.	Serum prolactin concentration at 15 and 30 minutes following intra venous TRH (200mcg) stimulation. Luteal phase length, number of days. Measurements on menstrual cycle days 5 to 8 and 20 for FSH, LH, oestradiol, progesterone, DHEAs, thyroid stimulating hormone (TSH), T3, T4, testosterone.	No significant changes in prolactin before and after in either group.	In this study 52 women were eligible to participate, statistical analyses were performed on data from 37 women.
					Length (number of days) of the luteal phase before and after; treatment group 3.4 (±5.0) to 10.5 (±4.3) (p < 0.005), placebo 3.4 (±5.1) to 5.5 (±5.2), p = 0.22.	There is missing data due to the presence of luteinised, unruptured follicles (9 women). These data were not included in analyses. Six women did not present for further investigation.
					Mid luteal (day 20) serum progesterone concentration before and after; treatment arm 2.46 (±0.70) to 9.69 (±6.34), p < 0.001. Placebo 1.99 (±0.65) to 2.34 (±0.59) p = 0.08.	No description of the distribution of drop-outs or missing data. This suggests the potential imbalance between intervention and control and a possible over-exaggeration for treatment effect.
					Mid-cycle oestradiol; treatment arm 131.6 (±25.0) to 151.6 (±25.4), p < 0.05. Placebo: 119.5 (±26.0) to 131.1 (±33.2) p = 0.22. Pregnancies in treatment group n = 2.	Intention to treat analysis was not performed.
						Unaccounted confounding factors include medications, fertility status, duration of latent hyperprolactinaemia.
Shahin et al. [65]	Randomised controlled trial using with an active control arm for comparative effectiveness. One menstrual cycle.	147 women aged less than 35 years with un-explained infertility and recurrent clomiphene resistance for ovulation induction. Anovulatory participants were excluded (n = 28). Anovulation was diagnosed by serum oestradiol < 200 ng/ml and absence of a dominant ovarian follicle on day 9 of the menstrual cycle. Complete data sets available for 119 women.	All women received Clomiphene citrate (clomiphene) 150 mg on menstrual cycle days 3–7. A randomised group also took *Cimicifuga racemosa* 20 mg per day between days 1–12. *Cimicifuga racemosa* described as ‘phytoestrogens’ was provided in the commercial preparation Klimadynon®, manufactured by Norica in Germany. A trigger injection (human chorionic gonadotropin, 10 000 IU) and timed intercourse was recommended when a dominant follicle > 17 mm was observed.	Pregnancy rate measured as increasing serum human chorionic gonadotropin (HCG) over two days. Clinical pregnancy defined as detection of gestational sac with embryonic heart-beat. Endometrial thickness measured by ultrasound concurrent with follicle maturation monitoring. Number of days to ovulation (trigger injection) Serum concentration for FSH oestradiol and LH. Luteal progesterone measured on days 21–23 of the menstrual cycle. Miscarriage and multiple pregnancy rates.	Pregnancy rate in clomiphene alone group was 20.3% and 43.3% in the clomiphene plus *Cimicifuga racemosa* group (P < 0.01). Clinical pregnancy rate in the combination group was 36.7% versus 13.6% in the clomiphene alone group (P < 0.01). Endometrial thickness in combination group was 8.9 (±1.4) versus 7.5 (±1.3) (p < 0.001). Days to ovulation in clomiphene alone group was 13.0 ± 1.1 and in the clomiphene plus *Cimicifuga racemosa* group 14.2 ± 1.3 (n.s.). Luteal progesterone peak (ng/ml) in combination group was 13.3 (±3.1) versus 9.3 (±2.0) in clomiphene alone group (p < 0.01). All other hormone measures were not significantly different	No detailed current baseline criteria for other causes of infertility. Confounding factors include current male fertility status. This may have caused an imbalance between the two groups. There is no description of the distribution of excluded (anovulatory) participants between groups.
Kamel [67]	Randomised controlled trial with an active control group. Comparative effectiveness trial for ovulation induction in women with PCOS. Three menstrual cycles.	Women aged 21–27 with primary or secondary infertility. Diagnosis of PCOS by ultrasound and clinical history (n = 100). Gynaecology outpatient clinic. Two groups. Group one (n = 50) received Clomiphene citrate 100 mg days 2–7 of the menstrual cycle; group two (n = 50) received 20 mg *Cimicifuga racemosa* for days 2–12 of the menstrual cycle.	*Cimicifuga racemosa* extract Klimadynon® by Bionorica, Neumarkt i.d. OBF Germany. 20 mg twice daily days 2–12 of menstrual cycle Clomiphene citrate (clomiphene) 100 mg daily for days 2–7 of menstrual cycle. Trigger injection (Human chorionic gonadotropin Pregnyl) and timed intercourse recommended when dominant follicle (>18 mm) was observed on ultrasound.	Serum measurements during follicular phase for FSH, LH and FSH:LH ratio. Mid luteal progesterone. Ultrasound observation of endometrial thickness. Pregnancy rates including twin pregnancies. Adverse events including hyperstimulation.	Positive outcomes for *Cimicifuga racemosa* compared to clomiphene for reduced day 2–5; LH (p = 0.007) and improved FSH to LH ratio (p = 0.06), mid luteal progesterone (p = 0.0001), endometrial thickness (p = 0.0004). Pregnancy rates were higher in the *Cimicifuga racemosa* group (7/50 compared to 4/50) but not statistically significant (p = 0.1). Adverse events (4 women) and twin pregnancy’s (two women) were not significantly different between groups.	No detail for diagnostic criteria for PCOS. Confounding fertility factors not described. Drop-out reasons were not reported seven in *Cimicifuga racemosa* group and four in clomiphene group.
Shahin [68]	Non-blinded randomised controlled trial.	Women with PCOS and infertility, n = 194.	All participants received pharmaceutical ovulation induction (Clomiphene citrate 150 mg on days 3–7 of cycle); trigger injection (HCG 10000 IU Pregnyl), timed intercourse and progesterone support (oral micronized progesterone). A randomly selected group additional took *Cimicifuga racemosa* 120 mg per day (Klimadynon®)	Primary outcomes pregnancy rates. Secondary outcomes:	Pregnancy rates were 33 out of 192 cycles (17.2%) for the clomiphene alone group and 71 out of 204 cycles (34.8%) for the clomiphene plus *Cimicifuga racemosa* group.	Non-blinding compromised the internal validity of the findings in this study. Confounding variables include variations in participant’s and clinicians attitudes and may have led to differences which were unaccounted for between the two groups. However the outcomes are objective with a statistically powered sample size.
	Three menstrual cycles each separated by two months of no treatment.	Two groups matched for demographics, age, BMI, primary and secondary infertility and duration of infertility (months). Treatment arm n = 96, control n = 98.		1. Number of days to ovulation (trigger injection). Follicular maturation monitored by ultrasound.	Number of days to trigger injection was 15 (±1.7) for the clomiphene alone group and 12.0 (±1.9) in the clomiphene plus *Cimicifuga racemosa* group (p = 0.01)	Measures for miscarriages are based on per cycle are not valid. Miscarriages per pregnancy are of greater relevance.
		Randomisation for 206 women 12 were excluded due to failure to respond (treatment group n = 7, control n = 5).		2. Endometrial thickness monitored by ultrasound.	Endometrial thickness in the clomiphene alone group was 8.5 mm (±1.9) compared to 12.9 (±2.3) in the clomiphene plus *Cimicifuga racemosa* group (p < 0.001).	The miscarriage rate per pregnancy for the clomiphene alone group was 5 out of 33 (15.2%) and 6 out of 71 (8.5%) in the clomiphene plus *Cimicifuga racemosa* group.
				3. Serum hormones during follicular phase oestradiol, LH and FSH. Luteal progesterone measured day 21–23 of the cycle.	Serum LH was 8.0 (±0.9) in the clomiphene group and 5.7 (±0.9) in the clomiphene plus *Cimicifuga racemosa* group (p < 0.001) and oestradiol was 228.3 (±30.2) in the clomiphene alone group and 299.5 (±38.9) \in the clomiphene plus *Cimicifuga racemosa* group (p = 0.01)	
				4. Pregnancy outcomes for early miscarriage.	Miscarriages were 5 out of 192 cycles in the clomiphene group and 6 out of 204 cycles in the clomiphene plus *Cimicifuga racemosa* group (n.s.).	
Wang et al. 2008 [66]	Double blinded, placebo controlled randomised trial (pilot). Eight weeks.	15 overweight women with oligo/amenorrhoea and polycystic ovaries on ultrasound. Mean body mass index 28.8 ± 1.3 kg/m2. Mean age 31.1 ± 2.0 years	*Cinnamomum cassia* extract 333 mg (Integrity Nutraceuticals International Sarasota, Florida) or placebo. One tablet three times per day.	Primary outcomes: Insulin resistance and sensitivity. Secondary outcomes oestradiol and testosterone concentration. Body mass index (BMI). Before and after treatment comparisons between randomised groups plus comparison between treatment group and normal ovulatory, normal weight women. Adverse events.	Improved insulin sensitivity (QUICKI) in the treatment group. 0.35 to 0.38, (7.7%) p < 0.03. Insulin resistance (HOMO-IR) significantly reduced in treatment group 2.57 to 1.43 (44.5%) p < 0.03. Controls no change insulin sensitivity or insulin resistance. No change in either group for BMI, testosterone and oestradiol. Differences between *Cinnamomum cassia* group and normal weight and ovulatory controls were not significant. (P < 0.17). No reported adverse reactions.	Small pilot study, the authors report that larger studies are required to confirm findings. Small sample size may explain non-significant comparison with normal weight and ovulating women. Reproductive outcomes were unchanged in this study however the duration of the study was insufficient to demonstrate reproductive changes.

## Results

### Laboratory studies

**Figure 1:**
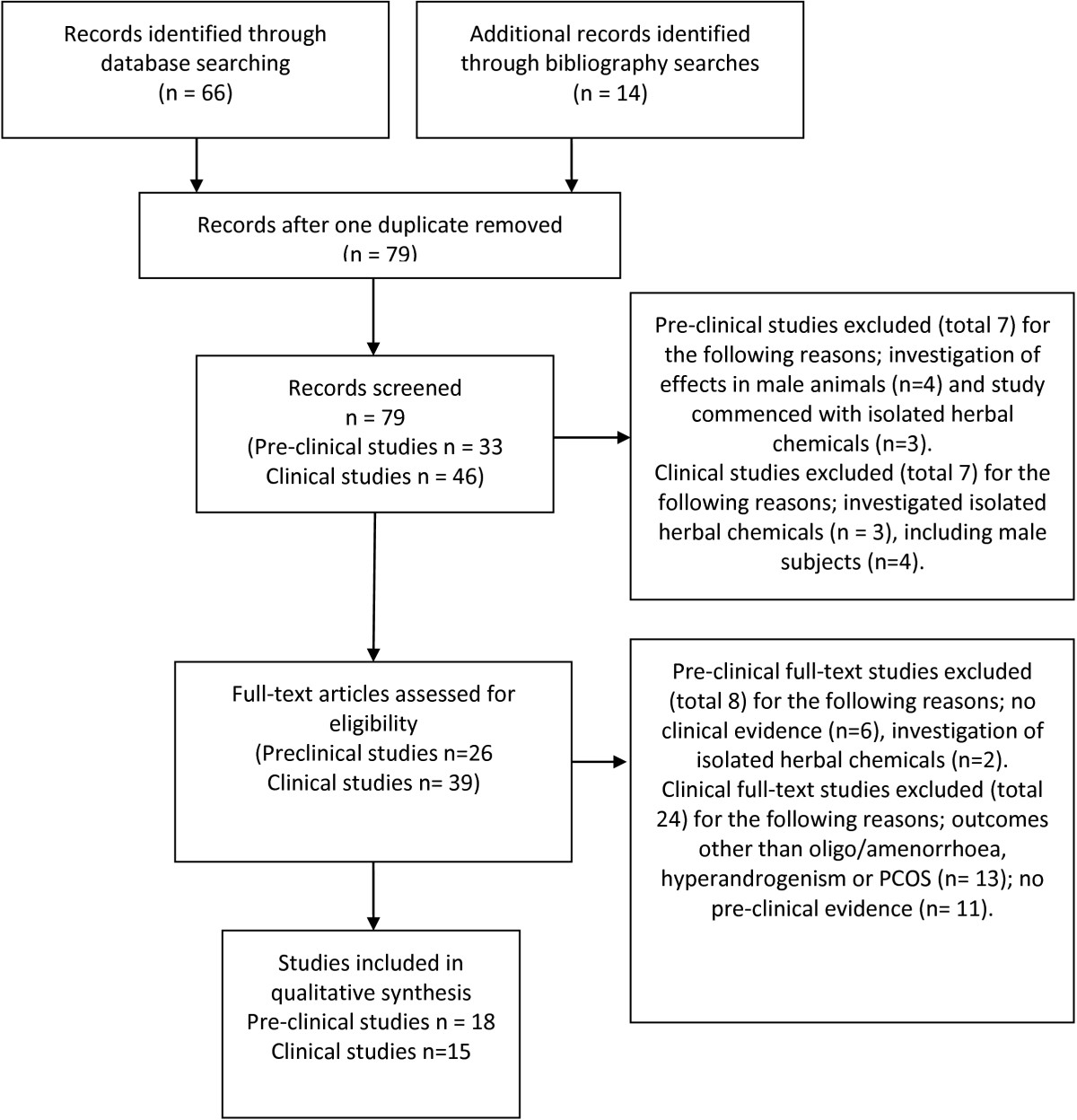
**Flow Chart.** Overarching results from two searches; preclinical data and clinical outcomes

Our search identified 33 laboratory (pre-clinical) studies (Figure 1). Eighteen studies met the inclusion criteria, nine reported on receptor binding assays or ovarian or pituitary (brain) cell cultures, [36–44] and nine used an animal experimental model with hormone assays and/or post-mortem examination of ovarian, uterine and brain histology, [45–53] (Table 1). We excluded 15 studies for the following reasons; investigation of effects in male animals (n = 4) and investigations which commenced with constituents that were isolated from herbal medicines (n = 5). Six studies were excluded due to no clinical evidence found (n = 6).

### Clinical studies

Following the electronic and manual searches of bibliographies, forty six clinical studies were identified for inclusion/exclusion assessment (Figure 1). A pre-requisite for the inclusion of clinical studies was identified laboratory evidence explaining the mechanism of effect in reproductive endocrinology. Fifteen met the inclusion criteria [54–68]. Eight were randomised controlled trials (RCTs) including 762 women [61–68] (Table 2). Thirty one studies were excluded for the following reasons; investigation of isolated herbal chemicals (n = 3); inclusion of male subjects (n = 4); no pre-clinical evidence (n = 11) and conditions different to those specified (n = 13).

### Excluded studies

Details of excluded studies investigating herbal medicines with clinical evidence but no preclinical evidence were provided in Table 3. Herbal medicines with preclinical evidence but no clinical evidence were provided in Table 4, and investigations into isolated chemicals derived from herbal medicine were presented in Table 5.

**Table 3:** **Herbal medicines with clinical evidence not included in this review**

Herbal medicine	Clinical evidence (or potential) for PCOS and associated oligo/amenorrhoea or hyperandrogenism	Reason for non-inclusion – insufficient pre-clinical evidence for mechanism of effects for whole herbal extract
*Camellia sinensis* (green tea)	Hormone concentration in obese women with PCOS [71].	Isolated constituent (epigallocatechin gallate 1) examined [72]. No evidence found for effects for whole herbal extract in PCOS, oligo/amenorrhoea and hyperandrogenism.
*Mentha spicata* (spearmint tea)	Lowered testosterone in women with PCOS [73, 74].	No evidence for mechanism of effect found for PCOS, oligo/amenorrhoea or hyperandrogenism.
*Ginkgo Biloba* (ginkgo)	Metabolic hormone management for type two diabetes [75].	No evidence for mechanism of effect in PCOS, oligo/amenorrhoea or hyperandrogenism found.
*Grifola frondosa* (miatake mushroom)	Ovulation rates in PCOS [76].	No evidence for mechanism of effect in PCOS, oligo/amenorrhoea or PCOS revealed.
*Linum usitatissimum* (flax seed)	Menstrual regulation [77, 78] and hormonal concentration [78–80] in post-menopausal women.	No mechanism of effect in PCOS, oligo/amenorrhoea or hyperandrogenism found.
*Pygeum africanum* (pygeum)	Anti-androgen effects in prostatic hypertrophy [81].	No evidence for mechanism of effect found in PCOS, oligo/amenorrhoea or hyperandrogenism (in female cell cultures or animals).
*Serrenoa repens* (saw palmetto)	Anti-androgen effects in chronic pelvic pain and prostatitis [82–84].	No mechanism of effect in PCOS, oligo/amenorrhoea or hyperandrogenism (in female cell cultures or animals).
*Silybum marianum* (St Mary’s thistle)	Fatty liver disease in type two diabetes [85].	No mechanism of effect in PCOS, oligo/amenorrhoea or hyperandrogenism.
*Stachys lavandulifolia* (wood betony)	Evidence for improved uterine bleeding (including oligomenorrhoea and amenorrhoea) in women with PCOS comparable with Medroxyprogesterone acetate [86].	No mechanism of effect studies found for whole herbal extract in PCOS and or associated oligo/amenorrhoea and hyperandrogenism.
*Urtica dioca* (nettle root)	Anti-androgen effects in women [87].	Anti-androgen effects through interaction with SHBG in prostate cells [88–90]. Anti-inflammatory and anti-nociceptive effects [91] No evidence for effects of *Urtica dioca* in female cell cultures or animals.

Other excluded studies investigated the herbal medicines included in this review examining conditions other than PCOS, oligo/amenorrhoea and hyperandrogenism. These included investigations into effectiveness for *Vitex agnus-castus* for pre-menstrual syndrome [92–97] and mastalgia [98, 99], *Cimicifuga racemosa* for menopausal symptoms [100] and Glycyrrhiza spp with Paeonia lactiflora libido in males [101].

**Table 4:** **Herbal medicines with pre-clinical evidence not included in this review**

Herbal medicine	Pre-clinical evidence for (potential) effects in reproductive endocrinology in PCOS and associated oligo/amenorrhoea and hyperandrogenism	Reason for exclusion
*Curcuma longa* (turmeric)	Anti-androgen effects [102].	No clinical evidence examining effectiveness in women was found.
*Matricaria chamomilla* (Chamomile)	Reduced luteinising hormone and improved ovarian morphology in animals with PCOS [103].	No clinical data found.
*Mentha piperita* (peppermint)	Anti-androgen effects in animals [104].	No clinical data for women.
*Silybum marianum* (St Marys thistle)	Anti-proliferative antioxidant and biochemical effects in the liver [105].	No clinical evidence including women was found.

Studies investigating chemical compounds derived from the herbal medicines, included in this review but investigating different outcomes were found for Vitex agnus-castus [70] and Cimicifuga racemosa [106].

**Table 5:** **Chemicals derived from herbal medicines not included in this review**

Isolated chemicals	Evidence for effects
Phytoestrogens	Hormonal effects in ovarian granulosa cells [107].
Berberine	Comparison with metformin in PCOS [16]; ovarian theca cell hormone production [108].
Catechin derived from *Camellia sinensis*	Effects of epigallocatechin gallate 1 on cellular metabolic endocrinology [72].
Sapponins derived from *Tribulus terrestris*	Effects on reproductive endocrinology [109].
Paeoniflorin, glycyrrhizin and glycyrrhetic acid	Ovarian androgen production [110].
Isoflavones isolated from *Vitex agnus-castus*	Selective oestrogen receptor activity (competitive inhibition via beta oestrogen receptors) [36].

Seven RCTs examined commercially produced herbal medicine extracts. These were *Vitex agnus-castus* in the form of Strontan®[64], Mastodynon®[61], Phyto Hypophyson®[62] and Agnacaston®[63] and *Cimicifuga racemosa* in the form of Klimadynon®[65, 67, 68] (Table 2).

### Herbal medicines with effects in oligo/amenorrhoea, hyperandrogenism and PCOS

The results of preclinical studies and clinical studies have been summarised together for each of the six herbal medicines.

### Vitex agnus-castus

Pre-clinical and clinical evidence was found for *Vitex agnus-castus* for lowered prolactin, improved menstrual regularity and treatment of infertility. *Vitex agnus-castus* contains a variety of compounds which bind to dopamine type 2 (DA-2) receptors in the brain; reduce cyclic adenosine mono phosphate (cAMP) and lowered prolactin secretion (Table 1). This was demonstrated in studies using recombinant DA-2 receptor proteins, and basal and stimulated rat pituitary cell cultures [38–41]. Prolactin lowering effects were found in normal and ovariectomised rats [49]. Additional agonistic opiate effects were observed in studies using human opiate receptors cell cultures [70].

Clinical equivalence for prolactin lowering effects of *Vitex agnus-castus* (Agnucaston® 40 mg per day) and the pharmaceutical Bromocriptine (Parlodel® 5 mg per day) was found in one study including 40 women with hyperprolactinaemia [63]. Mean concentrations for prolactin following three months treatment with *Vitex agnus-castus* was significantly reduced from 946 mIU/l (±173) to 529 mIU/l (±297) (p < 0.001). Comparatively, mean prolactin concentration in the Bromocriptine group was significantly reduced from 885 mIU/l (±178) to 473 mIU/l (±266) (p < 0.001) demonstrating that both treatments were effective treatment for women with hyperprolactinaemia (normal reference range 25-628 mIU/l). The mean difference in prolactin reduction of the two groups was not significant (p = 0.96) (Table 2).

Positive effects for V*itex agnus-castus* in oligo/amenorrhoea and infertility was demonstrated in three placebo controlled RCTs [61, 62, 64]. In a study including women with menstrual irregularity and infertility (n = 96), menstrual cyclicity was significantly improved for women treated with *Vitex agnus-castus* (Mastodynon® 30 drops per day for three months) compared to placebo (p = 0.023) [61] (Table 2). Another study, including women with sub fertility (n = 67), showed improved menstrual cyclicity for a sub-group of women with oligomenorrhoea following treatment with *Vitex agnus-castus* (Phyto-Hypophyson® 7.5 ml per day) compared to placebo, (p = 0.023) [62] (Table 2). A third study including women with hyperprolactinaemia (n = 37) demonstrated improved menstrual cyclicity by an increased average number of luteal days from 3.4 days (±5.0) to 10.5 days (±4.3) (p < 0.005) following treatment with *Vitex agnus-castus* (Strotan® 20 mg per day) for three months. The placebo group reported average number of days in the luteal phase was 3.4 (±5.1) at baseline and 5.5 (±5.2) at three months, which was not significant (p = 0.22) [64] (Table 2). Methodological shortcomings included not reporting baseline characteristics for subgroups and small sample sizes; however clinical outcomes demonstrated physiological effects consistent with laboratory and animal findings (Tables 1 and 2).

### Cimicifuga racemosa

*Cimicifuga racemosa* was found to lower LH in two laboratory studies both examining cell cultures from ovariectomised rats [45, 52] (Table 1). The mechanism occurred through competitive inhibition of oestrogen following the selective binding of oestrogen receptors (ERα) on the hypothalamus and pituitary [52]. An earlier study found contrary results for reduction of LH, however this study investigated an isolated flavonoid and suggested that other constituents may be active [37].

Three RCTs corroborate the positive fertility effects for *Cimicifuga racemosa* in women with PCOS, used in conjunction and when compared with the pharmaceutical Clomiphene citrate (clomiphene), [65, 68, 71] (Table 2). Results were reported for 441 women and show improved pregnancy rates when *Cimicifuga racemosa* was added to clomiphene during one menstrual cycle. In a study including women with PCOS (n = 147), pregnancy rates for the group receiving combined therapy (clomiphene 150 mg plus *Cimicifuga racemosa* 20 mg per day (Klimadynon®)) were 43.3% compared to 20.3% for women receiving only clomiphene [65] (Table 2). In another study using similar methodology (n = 100) pregnancy rates were 34.8% for the group treated with *Cimicifuga racemosa* plus clomiphene compared to 17.2% for women treated with clomiphene alone [68] (Table 2). Another study included women with PCOS and infertility (n = 100) compared Cimicifuga racemosa (Klimadynon®) and clomiphene over three months for hormone concentrations and pregnancy rates. Pregnancy rates were higher in the women in taking *Cimicifuga racemosa* compared to clomiphene, 14% and 8% respectively; however differences were not statistically significant. This study found significant effects for lowered luteinising hormone for women with PCOS receiving *Cimicifuga racemosa* compared to clomiphene (p = 0.007) [67]. Findings from clinical studies concur with laboratory and animal studies; however potential risks for bias include performance and collection bias due to lack of blinding (Table 2).

### Tribulus terrestris

Two laboratory based RCT’s examined the effects of *Tribulus Terrestris* in rats with polycystic ovaries induced with oestradiol valerate [46, 47] (Table 1). Both studies demonstrate significantly improved ovulation rates for animals treated with two doses of *Tribulus terrestris* extracts compared to controls. Although the endocrinological effects were not described in either study, laboratory findings of ovulation induction are supported by the clinical findings of elevated FSH following treatment with *Tribulus terrestris*[56] (Table 2).

A prospective, observational clinical trial examined the endocrine effects of *Tribulus terrestris* 750 mg per day, over five days in eight healthy women (aged 28–45). A significant increase in mean serum FSH concentration from 11 mIU/ml before treatment to 17.75 mIU/ml following treatment (P < 0.001) was demonstrated. Pre-treatment FSH levels returned following cessation of treatment (Table 1). Another clinical study evaluated the equivalence of *Tribulus terrestris* (Tribestan®) and pharmaceuticals for ovulation induction in women with oligo/anovular infertility (n = 148), [60]. During the three month follow up, ovulation rates were highest with epimestrol (74%), followed by *Tribulus terrestris* (60%), clomiphene (47%) and cyclofenil (24%). However, the evidence for *Tribulus terrestris* should be interpreted with caution due to risks for bias in clinical studies. One study was uncontrolled with a small number of healthy participants [56], the second study did not report baseline characteristics, methods for allocation to treatment groups and data were not statistically analysed [60] (Table 1).

### Glycyrrhiza spp

Androgen lowering effects for *Glycyrrhiza spp.* have been demonstrated in one laboratory study examining hormone concentration in female rats (*Glycyrrhiza uralensis*), [53] and corroborated in two clinical trials, one including healthy women [55] and the other including women with PCOS (*Glycyrrhiza glabra*) [54] (Table 1). The animal study reported significantly reduced free and total testosterone and increased oestradiol in sterilised rats and no hormonal changes in oophrectomised rats. The authors conclude that the hormonal effects occurred primarily in the ovary via enhanced aromatisation of testosterone to 17-beta oestradiol. The investigators also observed significantly increased oestradiol. There were no changes to FSH or LH in androgen sterilised or oophrectomised rats [53].

Another animal study examined the effects of *Glycyrrhiza uralensis* on the morphological features of polycystic ovaries using immunohistochemistry [50] (Table 1). This study demonstrated significantly increased ovulation rates by the number of corpus luteum in polycystic ovaries compared with controls. The authors propose that the mechanism of effect for *Glycyrrhiza uralensis* was competitive inhibition of oestrogen at oestrogen receptor sites, limiting the production of nerve growth factor (NGF), its neurotropic effects and inhibition of sympathetic neurological involvement in the pathogenesis of polycystic ovaries.

Two clinical studies examined the androgen lowering effects of *Glycyrrhiza Glabra*. A single arm clinical trial demonstrated reduced testosterone in healthy women aged 22–26 years (n = 9) over two menstrual cycles. Treatment with *Glycyrrhiza glabra,* 7 grams per day reduced testosterone from 27.8(±8.2) to 17.5 (±6.4), p < 0.05 [55]. Another single arm clinical trial investigated the effects of *Glycyrrhiza glabra* in women with PCOS, (n = 32). *Glycyrrhiza glabra* 3.5 g per day was added to anti-androgen pharmaceutical treatment, Spirinolactone 100 mg/day over two menstrual cycles. An unwanted side effect for Spirinolactone was the flare of androgens during the initial phase of treatment. This study demonstrated reduced concentrations of testosterone during the first four days of treatment at 103 ± 29 ng/d in the Spirinolactone group compared to 91 ng/d (±19) when combined with *Glycyrrhiza glabra* (p < 0.05) [54] (Table 1). Consistent laboratory and clinical outcomes were demonstrated however limitations included design shortcomings. Both clinical studies were open label observational design with small sample sizes; one included healthy participants. Rigorous studies are needed to confirm the androgen lowering effects of *Glycyrrhiza spp.* in hyperandrogenism and PCOS.

Results for *Glycyrrhiza Spp.* (and indeed any herbal ingredient) were complicated in this case by the variation in herbal extraction processes and subsequent variability in chemical profiles of the herbal ingredients. The laboratory studies of the herbal material were based on aqueous extracts of crude material whilst the clinical studies were based on ethanol extracts. Despite variability in the herbal extraction methods, both laboratory and clinical studies demonstrated anti-androgenic effects.

### *Paeonia lactiflora* and *glycyrrhiza uralensis*

One laboratory study and two clinical investigations provided evidence for the two herb combination, *Glycyrrhiza uralensis* and *Paeonia lactiflora*[53, 58, 59] (Table 1). An animal study found significant reductions in free and total testosterone following exposure to the combination [53] (Table 1). These findings were supported in two open label clinical trials including women with PCOS (n = 34) [59] and women with hyperandrogenism (n = 8) [58]. Both trials examined the effects on androgens for the aqueous extract TJ-68 (equal parts *Glycyrrhiza uralensis* and *Paeonia lactiflora*), 75 grams per day for 24 weeks and 5–10 grams per day for 2–8 weeks respectively. In the trial including women with PCOS, mean serum testosterone was significantly reduced from 137.1 ng/dL (±27.6) to 85.3 ng/dL (±38), p < 0.001 at four weeks of treatment [59]. Similar effects were observed in the women with oligomenorrhoea and hyperandrogenism which showed serum testosterone reduced from 50-160 ng/dL prior to treatment to less than 50 ng/dL [58]. However statistical significance was not reached due to the small sample size despite positive outcomes in seven out of eight participants (Table 1).

### *Paeonia lactiflora* and *cinnamomum cassia*

*Paeonia lactiflora* combined with *Cinnamomum cassia* in a preparation called Unkei-to was investigated in an in-vitro study for ovarian production of 17-beta-oestradiol and progesterone, [42] (Table 1). Granulosa cells obtained from women undergoing IVF were examined for steroid hormone concentration following incubation with different doses over 48 hours. Oestradiol was significantly increased (p < 0.01) following exposure to doses of 0.3 ug/ml of Unkei-to. Supporting clinical evidence was found in one clinical trial of 157 infertile women aged 17–29 years, including a subgroup of 42 women with hyper-functioning (PCOS) oligo/amenorrhoea. Treatment with Unkei-to, 7.5 grams per day for eight weeks, demonstrated significant reductions of mean LH in the PCOS sub-group of 49.7% (±15.3). Ovulation was confirmed in 30 out of 42 oligo/amenorrheic women [57] (Table 1). Limitations however include findings based on sub-group comparisons without description of subgroup baseline characteristics (other than oligomenorrhoea). Although the same aqueous extract intervention was investigated in pre-clinical and clinical studies, it contained additional herbal extracts and it was irrational to attribute hormonal effects to *Paeonia lactiflora* and *Cinnamomum cassia*.

### Cinnamomum cassia

An animal study compared the effectiveness of *Cinnamomum cassia* and the pharmaceutical Metformin on hormone concentration in rats with PCOS [48] (Table 1). Both interventions demonstrated significant improvements compared to controls at 15 days for measures of testosterone ng/ml (control 0.747 ± 0.039; metformin 0.647 ± 0.027; *Cinnamomum cassia* 0.625 ± 0.029); LH ng/ml (control 7.641 ± 0.267; metformin 6.873 ± 0.214; *Cinnamomum cassia* 6.891 ± 0.221) and insulin resistance (HOMA-IR) (control 10.018 ± 0.217; metformin 7.067 ± 0.184 *Cinnamomum cassia* 8.772 ± 0.196) (p < 0.05) [48]. The metabolic effects for *Cinnamomum cassia* were further demonstrated in overweight women with oligo/amenorrhoea and PCOS in a placebo controlled RCT [66] (Table 2). However, although the RCT had low risks for bias, it was a pilot study primarily investigating feasibility. Outcomes were promising for metabolic profile in PCOS however the sample size was small and the authors recommended further studies.

### Summary of results

This review includes 18 preclinical laboratory based studies and 15 clinical trials. We found reproductive endocrine effects in oligo/amenorrhoea, hyperandrogenism and/or PCOS for six herbal medicines. The quality of evidence, as determined by the volume of pre-clinical studies and the methodological quality of clinical trials, was highest for the herbal medicines *Vitex agnus-castus*, *Cimicifuga racemosa* and *Cinnamomum cassia*, for which there were laboratory and/or animal studies demonstrating endocrine mechanisms of action consistent with clinical outcomes shown in RCT’s with low risks for bias. However, replicated RCT data was only found for one herbal medicine, *Cimicifuga racemosa.*

Evidence for *Tribulus terrestris*, *Glycyrrhiza spp.* alone and in combination with *Paeonia lactiflora* and *Paeonia lactiflora* with *Cinnamomum cassia* was limited by the volume of laboratory and animal studies, with only one to two studies found for each herb or herbal combination. There was supporting clinical data, however many were small single arm, open label studies measuring endocrine effects in healthy women. Evidence for these herbal medicines is preliminary and in an emergent phase.

## Discussion

This review synthesises the evidence for mechanisms of effect for herbal medicine in oligo/amenorrhoea, hyperandrogenism and PCOS. Laboratory, animal and clinical studies demonstrate that the herbal medicines *Vitex agnus-castus*, *Cimicifuga racemosa* and *Tribulus terrestris* initiate endocrine effects in the pituitary as measured by lowered prolactin and LH and raised FSH. Four herbal medicines, *Tribulus terrestris*, *Glycyrrhiza spp.*, (alone and in combination with *Paeonia lactiflora*), *Paeonia lactiflora* (in combination with *Cinnamomum cassia*) and *Cinnamomum cassia* demonstrated morphological changes in polycystic ovaries and steroidogenesis, including reduced ovarian volume and cysts, lowered androgens, improved insulin sensitivity and increased oestradiol.

Clinical investigations found no adverse effects for the six herbal medicines included in this review (Table 2). A comparative study investigating the pharmaceutical Bromocriptine and the herbal medicine *Vitex agnus-castus* found no side effects associated *Vitex agnus-ca* stus compared to 12.5% of participants taking Bromocriptine reporting nausea and vomiting [63]. No studies comparing the effectiveness for herbal medicines and the oral contraceptive pill in PCOS, oligo/amenorrhoea and hyperandrogenism were found.

Herbal medicine may present a treatment option for women with oligo/amenorrhoea, hyperandrogenism and PCOS as an adjunct or alternative treatment to pharmaceuticals with a high degree of acceptability by women with PCOS [6]. Preliminary evidence for equivalent treatment effects were found for the two pharmaceuticals and three herbal medicines. These were bromocriptine, in the management of hyperprolactinaemia and*Vitex agnus-castus* and clomiphene for infertility and ovulation induction and *Cimicifuga racemosa* and *Tribulus terrestris*. Herbal medicine had positive adjunct effects with the pharmaceuticals Spirinolactone in the management of hyperandrogenism (*Glycyrrhiza Spp.*), and clomiphene for PCOS related infertility (*Cimicifuga racemosa*). It is important however to highlight that evidence was provided by a limited number of clinical studies, some with significant risks for bias; particularly *Tribulus terrestris*, *Glycyrrhiza glabra* alone and in combination with *Paeonia lactiflora* and *Paeonia lactiflora* in combination with *Cinnamomum cassia*.

Selection of herbal medicines for the management of PCOS often includes the combined prescription of *Glycyrrhiza spp.* and *Paeonia lactiflora*[72–75]. We found preliminary evidence for this combination for hyperandrogenism only, and the evidence was more robust for *Glycyrrhiza spp.* alone than when combined with *Paeonia lactiflora*. Comparatively, our findings for the combination of *Peaonia lactiflora* and *Cinnamomum cassia* demonstrated no change in androgen concentration, suggesting that the anti-androgen activity in the *Glycyrrhiza spp.* and *Paeonia lactiflora* combination more likely attributable to *Glycyrrhiza spp.* However our findings may be complicated by the aqueous extraction methods used in the *Paeonia lactiflora* and *Cinnamomum cassia* combination and the preclinical studies into the *Glycorrhizza spp* and *Paeonia lactiflora* combination. More research into the anti-androgen effects of the combination *Glycyrrhiza spp.* and *Paeonia lactiflora* is needed to clarify the anti-androgen mechanism particularly if this herbal combination remains cornerstone herbal management for hyperandrogenism.

This review has some limitations. We used a methodological approach which was deductive and not consistent with traditional rationale for herbal selection. Our inclusion criteria for clinical studies were specific and relied upon our identification of herbal medicines with preclinical (laboratory based) evidence explaining the mechanisms of reproductive endocrinological effects in oligo/amenorrhoea, hyperandrogenism and PCOS. Clinical studies were excluded from this review due to the absence of evidence for whole herbal extracts. This was the case for *Camellia sinensis* (green tea) for which only one laboratory study investigated the effects of injecting epigallocatechin, a catechin found in green tea in animals [76]. High quality clinical evidence for *Camellia sinensis* was not presented in this review due to the absence of pre-clinical data explaining the mechanism for effect for the whole herbal extract [77]. *Mentha spicata* (spearmint) was another herbal medicine excluded from this review despite the availability of high quality clinical evidence demonstrating testosterone lowering effects in women with PCOS [78]. We found no laboratory evidence describing the mechanism of action for *Mentha spicata* in hyperandrogenism. *Camilla sinensis* and *Mentha spicata* are examples of herbal medicines excluded from this review due to not meeting the inclusion criteria. Studies investigating western herbal medicines excluded from this review are provided in Tables 3, 4 and 5.

Our search strategy may have restricted access due to limited search terms. We didn’t include alternative spelling of oestrogen and additional search terms for herbal medicine could have been included to increase sensitivity of the search.

This study synthesises the evidence for reproductive endocrine effects for six whole herbal medicine extracts that may be used to treat PCOS and associated oligo/amenorrhoea and hyperandrogenism. The findings were intended to add to clinicians understanding for the mechanisms of action for herbal medicine for treatment in these common conditions and reveal herbal medicines with reproductive endocrinological effects, currently demonstrated in scientific literature.

## Conclusions

Preclinical and clinical studies provide preliminary evidence that six herbal medicines may have beneficial effects for women with oligo/amenorrhea, hyperandrogenism and PCOS. The quality of the evidence is variable and strongest for *Vitex agnus-castus* and *Cimicifuga racemosa* in the management of oligo/amenorrhea and infertility associated with PCOS; and *Cinnamomum cassia* for improving metabolic hormones in PCOS. Evidence for *Tribulus terrestris*, *Glycyrrhiza spp.* alone and in combination with *Paeonia lactiflora* and *Paeonia lactiflora* combined with *Cinnamon cassia* is promising but in an emergent phase. Further investigations into the mechanisms of effect for herbal extracts are needed to complete our understanding of the reproductive endocrinological effects for herbal medicine for these common conditions.

## Authors’ information

SA is a doctoral research student and CAS, JAB and AB are supervisory personnel. The submission processing fee was provided by the University of Western Sydney as part of an academic institutional membership.

## Authors’ original submitted files for images

Below are the links to the authors’ original submitted files for images. Authors’ original file for figure 1

## References

[1] March WA, Moore VM, Willson KJ, Phillips DI, Norman RJ, Davies MJ (2010). The prevalence of polycystic ovary syndrome in a community sample assessed under contrasting diagnostic criteria. Hum Reprod.

[2] Teede HJ, Misso ML, Deeks AA, Moran LJ, Stuckey BG, Wong JL, Norman RJ, Costello MF, Guideline Development Groups (2011). Assessment and management of polycystic ovary syndrome: summary of an evidence-based guideline. Med J Aust.

[3] ESHRE (2012). Consensus on women’s health aspects of polycystic ovary syndrome (PCOS). Hum Reprod.

[4] Messinis IE (2005). Ovulation induction: a mini review. Hum Reprod.

[5] Tang T, Lord JM, Norman RJ, Yasmin E, Balen AH (2010). Insulin-sensitising drugs (metformin, rosiglitazone, pioglitazone, D-chiro-inositol) for women with polycystic ovary syndrome, oligo amenorrhoea and subfertility. Cochrane Database Syst Rev.

[6] Sills ES, Perloe M, Tucker MJ, Kaplan CR, Genton MG, Schattman GL (2001). Diagnostic and treatment characteristics of polycystic ovary syndrome: descriptive measurements of patient perception and awareness from 657 confidential self-reports. BMC Womens Health.

[7] Holden S, Davis R, Yeh G (2014). Pregnant Women’s Use of Complementary & Alternative Medicine in the United States. J Alternative Compl Med.

[8] Lunny CA, Fraser SN (2010). The Use of Complementary and Alternative Medicines Among a Sample of Canadian Menopausal‒Aged Women. J Midwifery Womens Health.

[9] Bishop JL, Northstone K, Green JR, Thompson EA (2011). The use of complementary and alternative medicine in pregnancy: data from the Avon Longitudinal Study of Parents and Children (ALSPAC). Complement Ther Med.

[10] Nordeng H, Bayne K, Havnen GC, Paulsen BS (2011). Use of herbal drugs during pregnancy among 600 Norwegian women in relation to concurrent use of conventional drugs and pregnancy outcome. Complement Ther Clin Pract.

[11] Smith CA, Bateson DJ, Weisberg E (2013). A survey describing the use of complementary therapies and medicines by women attending a family planning clinic. BMC Complement Altern Med.

[12] Stankiewicz M, Smith C, Alvino H, Norman R (2007). The use of complementary medicine and therapies by patients attending a reproductive medicine unit in South Australia: a prospective survey. Aust New Zeal J Obstet Gynaecol.

[13] Ren MQ, Kuhn G, Wegner J, Chen J (2001). Isoflavones, substances with multi-biological and clinical properties. Eur J Nutr.

[14] Whitten PL, Naftolin F (1998). Reproductive actions of phytoestrogens. Baillieres Clin Endocrinol Metab.

[15] Wolff MS, Teitelbaum SL, Pinney SM, Windham G, Liao L, Biro F, Kushi LH, Erdmann C, Hiatt RA, Rybak ME, Calafat AM (2010). Investigation of relationships between urinary biomarkers of phytoestrogens, phthalates, and phenols and pubertal stages in girls. Environ Health Perspect.

[16] Wei W, Zhao H, Wang A, Sui M, Liang K, Deng H, Ma Y, Zhang Y, Zhang H, Guan Y (2012). A clinical study on the short-term effect of berberine in comparison to metformin on the metabolic characteristics of women with polycystic ovary syndrome. Eur J Endocrinol.

[17] Francis G, Kerem Z, Makkar HPS, Becker K (2002). The biological action of saponins in animal systems: a review. Br J Nutr.

[18] Grant P, Ramasamy S (2012). An Update on Plant Derived Anti-Androgens. Int J Endocrinol Metabol.

[19] Norman RJ, Dewailly D, Legro RS, Hickey TE (2007). Polycystic ovary syndrome. Lancet.

[20] ESHRE (2008). Consensus on infertility treatment related to polycystic ovary syndrome. Hum Reprod.

[21] Brown J, Farquhar C, Beck J, Boothroyd C, Hughes E (2009). Clomiphene and anti-oestrogens for ovulation induction in PCOS. Cochrane Database Syst Rev.

[22] Polson D, Kiddy DS, Mason HD, Franks S (1989). Induction of ovulation with clomiphene citrate in women with polycystic ovary syndrome: the difference between responders and nonresponders. Fertil Steril.

[23] Kousta E, White D, Franks S (1997). Modern use of clomiphene citrate in induction of ovulation. Hum Reprod Update.

[24] Tang T, Glanville J, Hayden CJ, White D, Barth JH, Balen AH (2006). Combined lifestyle modification and metformin in obese patients with polycystic ovary syndrome. A randomized, placebo-controlled, double-blind multicentre study. Hum Reprod.

[25] Williamson E (2001). Synergy and other interactions in phytomedicines. Phytomedicine.

[26] Mills S, Bone K (2000). Principles and Practice of Phytotherapy.

[27] Wardle JL, Adams J, Lui C-W (2010). A qualitative study of naturopathy in rural practice: A focus upon naturopaths’ experiences and perceptions of rural patients and demands for their services. BMC Health Serv Res.

[28] Steel A, Wardle J, Diezel H, Johnstone K, Adams J (2014). Educating for collaboration: The outcomes of an interprofessional education workshop for complementary and alternative maternity care providers. Adv Integr Med.

[29] Teede H, Gibson-Helm M, Norman RJ, Boyle J (2013). Polycystic Ovary Syndrome: Perceptions and Attitudes of Women and Primary Health Care Physicians on Features of PCOS and Renaming the Syndrome. J Clin Endocrinol Metabol.

[30] ESHRE (2004). Revised 2003 consensus on diagnostic criteria and long-term health risks associated with polycystic ovary syndrome. Fertil Steril.

[31] Legro RS, Zaino RJ, Demers LM, Kunselman AR, Gnatuk CL, Williams NI, Dodson WC (2007). The effects of metformin and rosiglitazone, alone and in combination, on the ovary and endometrium in polycystic ovary syndrome. Am J Obstet Gynecol.

[32] Conway G, Honour J, Jacobs H (1989). Heterogeneity of the polycystic ovary syndrome: clinical, endocrine and ultrasound features in 556 patients. Clin Endocrinol (Oxf).

[33] Balen AH, Tan SL, Jacobs HS (1993). Hypersecretion of luteinising hormone: a significant cause of infertility and miscarriage. BJOG.

[34] Legro RS, Kunselman AR, Dodson WC, Dunaif A (1999). Prevalence and predictors of risk for type 2 diabetes mellitus and impaired glucose tolerance in polycystic ovary syndrome: a prospective, controlled study in 254 affected women. J Clin Endocrinol Metabol.

[35] Luciano A, Chapler F, Sherman B (1984). Hyperprolactinemia in polycystic ovary syndrome. Fertil Steril.

[36] Jarry H, Spengler B, Porzel A, Schmidt J, Wuttke W, Christoffel V (2003). Evidence for estrogen receptor beta-selective activity of Vitex agnus-castus and isolated flavones. Planta Med.

[37] Jarry H, Harnischfeger G, Düker E (1985). Studies on the endocrine effects of the contents of Cimicifuga racemosa. In vitro binding of compounds to estrogen receptors. Planta Med.

[38] Jarry H, Spengler B, Wuttke W, Christoffel V (2006). In vitro assays for bioactivity-guided isolation of endocrine active compounds in Vitex agnus-castus. Maturitas.

[39] Jarry H, Leonhardt S, Gorkow C, Wuttke W (2009). In vitro prolactin but not LH and FSH release is inhibited by compounds in extracts of Agnus castus: direct evidence for a dopaminergic principle by the dopamine receptor assay. Exp Clin Endocrinol Diabetes.

[40] Meier B, Berger D, Hoberg E, Sticher O, Schaffner W (2000). Pharmacological activities of Vitex agnus-castus extracts in vitro. Phytomedicine.

[41] Sliutz G, Speiser P, Schultz AM, Spona J, Zeillinger R (1993). Agnus-castus extracts inhibit prolactin secretion of rat pituitary cells. Horm Metab Res.

[42] Sun WS, Imai A, Tagami K, Sugiyama M, Furui T, Tamaya T (2004). In vitro stimulation of granulosa cells by a combination of different active ingredients of unkei-to. Am J Chin Med.

[43] Wuttke W, Jarry H, Christoffel V, Spengler B, Seidlová-Wuttke D (2003). Chaste tree (Vitex agnus-castus). Pharmacology and clinical indications. Phytomedicine.

[44] Zierau O, Bodinet C, Kolba S, Wulf M, Vollmer G (2002). Antiestrogenic activities of Cimicifuga racemosa extracts. J Steroid Biochem Mol Biol.

[45] Düker EM, Kopanski L, Jarry H, Wuttke W (1991). Effects of extracts from Cimicifuga racemosa on gonadotropin release in menopausal women and ovariectomized rats. Planta Med.

[46] Dehghan A, Esfandiari A, Bigdeli SM (2012). Alternative Treatment of Ovarian Cysts with Tribulus terrestris Extract: A Rat Model. Reprod Domest Anim.

[47] Esfandiari A, Dehghan A, Sharifi S, Najafi B, Vesali E (2011). Effect of Tribulus terrestris extract on ovarian activity in immature Wistar rat: a histological evaluation. J Anim Vet Adv.

[48] Heibashy M, Mazen G, Shahin M (2013). Metabolic Changes and Hormonal Disturbances in Polycystic Ovarian Syndrome Rats and the Amelioration Effects of Metformin and/or Cinnamon Extraction. J Am Sci.

[49] Ibrahim N, Shalaby AS, Farag RS, Elbaroty GS, Nofal SM, Hassan EM (2008). Gynecological efficacy and chemical investigation of Vitex agnus-castus L. fruits growing in Egypt. Nat Prod Res.

[50] Lee JC, Pak SC, Lee SH, Lim SC, Bai YH, Jin CS, Kim JS, Na CS, Bae CS, Oh KS (2003). The effect of herbal medicine on nerve growth factor in estradiol valerate-induced polycystic ovaries in rats. Am J Chin Med.

[51] Martino-Andrade AJ, Morais RN, Spercoski KM, Rossi SC, Vechi MF, Golin M, Lombardi NF, Greca CS, Dalsenter PR (2010). Effects of Tribulus terrestris on endocrine sensitive organs in male and female Wistar rats. J Ethnopharmacol.

[52] Seidlova-Wuttke D, Hesse O, Jarry H, Christoffel V, Spengler B, Becker T, Wuttke W (2003). Evidence for selective estrogen receptor modulator activity in a black cohosh (Cimicifuga racemosa) extract: comparison with estradiol-17beta. Eur J Endocrinol.

[53] Takeuchi T, Nishii O, Okamura T, Yaginuma T (1989). Effect of traditional herbal medicine, shakuyaku-kanzo-to on total and free serum testosterone levels. Am J Chin Med.

[54] Armanini D, Castello R, Scaroni C, Bonanni G, Faccini G, Pellati D, Bertoldo A, Fiore C, Moghetti P (2007). Treatment of polycystic ovary syndrome with spironolactone plus licorice. Eur J Obstet Gynecol Reprod Biol.

[55] Armanini D, Mattarello MJ, Fiore C, Bonanni G, Scaroni C, Sartorato P, Palermo M (2004). Licorice reduces serum testosterone in healthy women. Steroids.

[56] Milanov S, Maleeva A, Tashkov M (1981). Tribestan effect on the concentration of some hormones in the serum of healthy subjects.

[57] Ushiroyama T, Ikeda A, Sakai M, Hosotani T, Suzuki Y, Tsubokura S, Ueki M (2001). Effects of unkei-to, an herbal medicine, on endocrine function and ovulation in women with high basal levels of luteinizing hormone secretion. J Reprod Med.

[58] Yaginuma TI, Yasui R, Arai H, Kawabata T (1982). Effect of traditional herbal medicine on serum testosterone levels and its induction of regular ovulation in hyperandrogenic and oligomenorrheic women. Nippon Sanka Fujinka Gakkai Zasshi.

[59] Takahashi K, Kitao M (1994). Effect of TJ-68 (shakuyaku-kanzo-to) on polycystic ovarian disease. Int J Fertil Menopausal Stud.

[60] Tabakova P, Dimitrov M, Tashkov B (1984). Clinical studies on the preparation Tribestan in women with endocrine infertility or menopausal syndrome.

[61] Gerhard I, Patek A, Monga B, Blank A, Gorkow C (1998). Mastodynon® for Female Infertility. Randomized placebo controlled, clinical double-blind study. Forschende Komplementärmedizin/Res Compl Med.

[62] Bergmann J, Luft B, Boehmann S, Runnebaum B, Gerhard I (2000). The efficacy of the complex medication Phyto-Hypophyson L in female, hormone-related sterility. A randomized, placebo-controlled clinical double-blind study. Forschende Komplementärmedizin und klassische Naturheilkunde. Res Compl Nat Classical Med.

[63] Kilicdag E, Tarim E, Bagis T, Erkanli S, Aslan E, Ozsahin K, Kuscu E (2004). Fructus agni casti and bromocriptine for treatment of hyperprolactinemia and mastalgia. Int J Gynecol Obstet.

[64] Milewicz A, Gejdel E, Sworen H, Sienkiewicz K, Jedrzejak J, Teucher T, Schmitz H (1993). Vitex agnus castus extract in the treatment of luteal phase defects due to latent hyperprolactinemia. Results of a randomized placebo-controlled double-blind study. Arzneimittel-Forschung (Drug Res).

[65] Shahin AY, Ismail AM, Zahran KM, Makhlouf AM (2008). Adding phytoestrogens to clomiphene induction in unexplained infertility patients – a randomized trial. Reprod Biomed Online.

[66] Wang JG, Anderson RA, Graham GM, Chu MC, Sauer MV, Guarnaccia MM, Lobo RA (2007). The effect of cinnamon extract on insulin resistance parameters in polycystic ovary syndrome: a pilot study. Fertil Steril.

[67] Kamel HH (2013). Role of phyto-oestrogens in ovulation induction in women with polycystic ovarian syndrome. Eur J Obstet Gynecol Reprod Biol.

[68] Shahin AY, Mohammed SA (2014). Adding the phytoestrogen Cimicifugae Racemosae to clomiphene induction cycles with timed intercourse in polycystic ovary syndrome improves cycle outcomes and pregnancy rates-a randomized trial. Gynecol Endocrinol.

[69] Jarry H, Metten M, Spengler B, Christoffel V, Wuttke W (2003). In vitro effects of the Cimicifuga racemosa extract BNO 1055. Maturitas.

[70] Webster D, Lu J, Chen SN, Farnsworth NR, Wang ZJ (2006). Activation of the μ-opiate receptor by Vitex agnus-castus methanol extracts: Implication for its use in PMS. J Ethnopharmacol.

[71] Chan CC, Koo MW, Ng EH, Tang OS, Yeung WS, Ho PC (2006). Effects of Chinese green tea on weight, and hormonal and biochemical profiles in obese patients with polycystic ovary syndrome—a randomized placebo-controlled trial. J Soc Gynecol Investig.

[72] Kao Y-H, Hiipakka RA, Liao S (2000). Modulation of endocrine systems and food intake by green tea epigallocatechin gallate 1. Endocrinology.

[73] Akdoğan M, Tamer MN, Cüre E, Cüre MC, Köroğlu BK, Delibaş N (2007). Effect of spearmint (Mentha spicata Labiatae) teas on androgen levels in women with hirsutism. Phytother Res.

[74] Grant P (2010). Spearmint herbal tea has significant anti-androgen effects in polycystic ovarian syndrome. A randomized controlled trial. Phytother Res.

[75] Kudolo GB, Wang W, Javors M, Blodgett J (2006). The effect of the ingestion of Ginkgo biloba extract (EGb 761) on the pharmacokinetics of metformin in non-diabetic and type 2 diabetic subjects–A double blind placebo-controlled, crossover study. Clin Nutr.

[76] Chen J-T, Tominaga K, Sato Y, Anzai H, Matsuoka R (2010). Maitake mushroom (Grifola frondosa) extract induces ovulation in patients with polycystic ovary syndrome: a possible monotherapy and a combination therapy after failure with first-line clomiphene citrate. J Alternative Compl Med.

[77] Phipps WR, Martini MC, Lampe JW, Slavin JL, Kurzer MS (1993). Effect of flax seed ingestion on the menstrual cycle. (J Clin Endocrinol Metabo.

[78] Lampe J, Martini MC, Kurzer MS, Adlercreutz H, Slavin JL (1994). Urinary lignan and isoflavonoid excretion in premenopausal women consuming flaxseed powder. Am J Clin Nutr.

[79] Hutchins AM, Martini MC, Olson BA, Thomas W, Slavin JL (2001). Flaxseed consumption influences endogenous hormone concentrations in postmenopausal women. Nutr Cancer.

[80] Frische EJ, Hutchins AM, Martini MC, Thomas W, Slavin JL (2003). Effect of flaxseed and wheat bran on serum hormones and lignan excretion in premenopausal women. J Am Coll Nutr.

[81] Melo E, Bertero EB, Rios LA, Mattos D (2002). Evaluating the efficiency of a combination of Pygeum africanum and stinging nettle (Urtica dioica) extracts in treating benign prostatic hyperplasia (BPH): double-blind, randomized, placebo controlled trial. Int Braz J Urol.

[82] Yang J, Te AE (2005). Saw palmetto and finasteride in the treatment of category-III prostatitis/chronic pelvic pain syndrome. Curr Urol Rep.

[83] Morgia G, Mucciardi G, Madonia M, Castelli T, Favilla V, Magno C (2008). Treatment of chronic prostatitis/chronic pelvic pain syndrome (CP/CPPS) with Serenoa repens plus selenio and licopene (Profluss®): a randomized multicenter placebo-controlled study. J Urol.

[84] Casner PR, Bent S, Kane C, Shinohara K (2006). Saw palmetto for benign prostatic hyperplasia. N Engl J Med.

[85] Huseini HF, Larijani B, Heshmat R, Fakhrzadeh H, Radjabipour B, Toliat T, Raza M (2006). The efficacy of Silybum marianum (L.) Gaertn. (silymarin) in the treatment of type II diabetes: a randomized, double‒blind, placebo‒controlled, clinical trial. Phytother Res.

[86] Jalilian N, Modarresi M, Rezaie M, Ghaderi L, Bozorgmanesh M (2013). Phytotherapeutic Management of Polycystic Ovary Syndrome: Role of Aerial Parts of Wood Betony (Stachys lavandulifolia). Phytother Res.

[87] Najafipour F, Rahimi AO, Mobaseri M, Agamohamadzadeh N, Nikoo A, Aliasgharzadeh A (2014). Therapeutic effects of stinging nettle (Urtica dioica) in women with Hyperandrogenism. Int J Current Res Acad Rev.

[88] Hryb D, Khan MS, Romas NA, Rosner W (1995). The effect of extracts of the roots of the stinging nettle. Planta Med.

[89] Schöttner M, Ganßer D, Spiteller G (1997). Lignans from the Roots of Urtica spp. Planta Med.

[90] Zhang Q, Li L, Liu L, Li Y, Yuan L, Song L, Wu Z (2008). Effects of the polysaccharide fraction of Urtica fissa on castrated rat prostate hyperplasia induced by testosterone propionate. Phytomedicine.

[91] Hajhashemi V, Klooshani V (2013). Antinociceptive and anti-inflammatory effects of Urtica dioica leaf extract in animal models. Avicenna J Phytomedicine.

[92] Loch E, Böhnert K, Peeters M (1991). The treatment of menstrual disorders with Vitex agnus-castus tincture. Der Frauenarzt.

[93] Loch E, Selle H, Boblitz N (2000). Treatment of premenstrual syndrome with a phytopharmaceutical formulation containing Vitex agnus castus. J Womens Health Gend Based Med.

[94] Ma L, Lin S, Chen R, Zhang Y, Chen F, Wang X (2010). Evaluating therapeutic effect in symptoms of moderate-to-severe premenstrual syndrome with Vitex agnus castus (BNO 1095) in Chinese women. Aust New Zeal J Obstet Gynaecol.

[95] Schellenberg R (2001). Treatment for the premenstrual syndrome with agnus castus fruit extract: prospective, randomised, placebo controlled study. BMJ (Clin Res ed).

[96] Lauritzen CH, Reuter HD, Repges R, Böhnert KJ, Schmidt U (1997). Treatment of premenstrual tension syndrome with Vitex agnus castus controlled, double-blind study versus pyridoxine. Phytomedicine.

[97] He Z, Chen R, Zhou Y, Geng L, Zhang Z, Chen S, Yao Y, Lu J, Lin S (2009). Treatment for premenstrual syndrome with Vitex agnus castus: A prospective, randomized, multi-center placebo controlled study in China. Maturitas.

[98] Wuttke W, Splitt G, Gorkow C, Sieder C (1997). Treatment of cyclical mastalgia: Results of a randomised, placebo- controlled, double-blind study: Objective. Obstet Gynecol.

[99] Halaska M, Beles P, Gorkow C, Sieder C (1999). Treatment of cyclical mastalgia with a solution containing a Vitex agnus castus extract: results of a placebo-controlled double-blind study. Breast.

[100] Wuttke W, Seidlova-Wuttke D, Gorkow C (2003). The Cimicifuga preparation BNO 1055 vs. conjugated estrogens in a double-blind placebo-controlled study: effects on menopause symptoms and bone markers. Maturitas.

[101] Yamada K, Kanba S, Yagi G, Asai M (1999). Herbal medicine (Shakuyaku-kanzo-to) in the treatment of risperidone-induced amenorrhea. J Clin Psychopharmacol.

[102] Ohtsu H, Xiao Z, Ishida J, Nagai M, Wang HK, Itokawa H, Su CY, Shih C, Chiang T, Chang E (2002). Antitumor agents. 217. Curcumin analogues as novel androgen receptor antagonists with potential as anti-prostate cancer agents. J Med Chem.

[103] Zangeneh FZ, Minaee B, Amirzargar A, Ahangarpour A, Mousavizadeh K (2010). Effects of chamomile extract on biochemical and clinical parameters in a rat model of polycystic ovary syndrome. J Reprod Infertil.

[104] Akdogan M, Ozguner M, Kocak A, Oncu M, Cicek E (2004). Effects of peppermint teas on plasma testosterone, follicle-stimulating hormone, and luteinizing hormone levels and testicular tissue in rats. Urology.

[105] Gebhardt R (2003). Antioxidative, antiproliferative and biochemical effects in HepG2 cells of a homeopathic remedy and its constituent plant tinctures tested separately or in combination. Arzneimittel Forschung.

[106] Burdette JE, Liu J, Chen S, Fabricant DS, Piersen CE, Barker EL, Pezzuto JM, Mesecar A, van Breemen RB, Farnsworth NR (2003). Black cohosh acts as a mixed competitive ligand and partial agonist of the serotonin receptor. J Agric Food Chem.

[107] Whitehead SA, Lacey M (2003). Phytoestrogens inhibit aromatase but not 17β hydroxysteroid dehydrogenase (HSD) type 1 in human granulosa luteal cells: evidence for FSH induction of 17β HSD. Hum Reprod.

[108] Zhao L, Li W, Han F, Hou L, Baillargeon J-P, Kuang H, Wang Y, Wu X (2011). Berberine reduces insulin resistance induced by dexamethasone in theca cells in vitro. Fertil Steril.

[109] Kostova I, Dinchev D (2005). Saponins in Tribulus terrestris - Chemistry and bioactivity. Phytochem Rev.

[110] Takeuchi T, Nishii O, Okamura T, Yaginuma T (1991). Effect of paeoniflorin, glycyrrhizin and glycyrrhetic acid on ovarian androgen production. Am J Chin Med.

[CR111] The pre-publication history for this paper can be accessed here:http://www.biomedcentral.com/1472-6882/14/511/prepub

