# Supplementary figures and images for: Herbal medicine for the management of polycystic ovary syndrome (PCOS) and associated oligo/amenorrhoea and hyperandrogenism; a review of the laboratory evidence for effects with corroborative clinical findings
Source: BMC Complement Altern Med. 2014 Dec 18;14:511. doi: 10.1186/1472-6882-14-511 (PMC4528347; doi:10.1186/1472-6882-14-511)

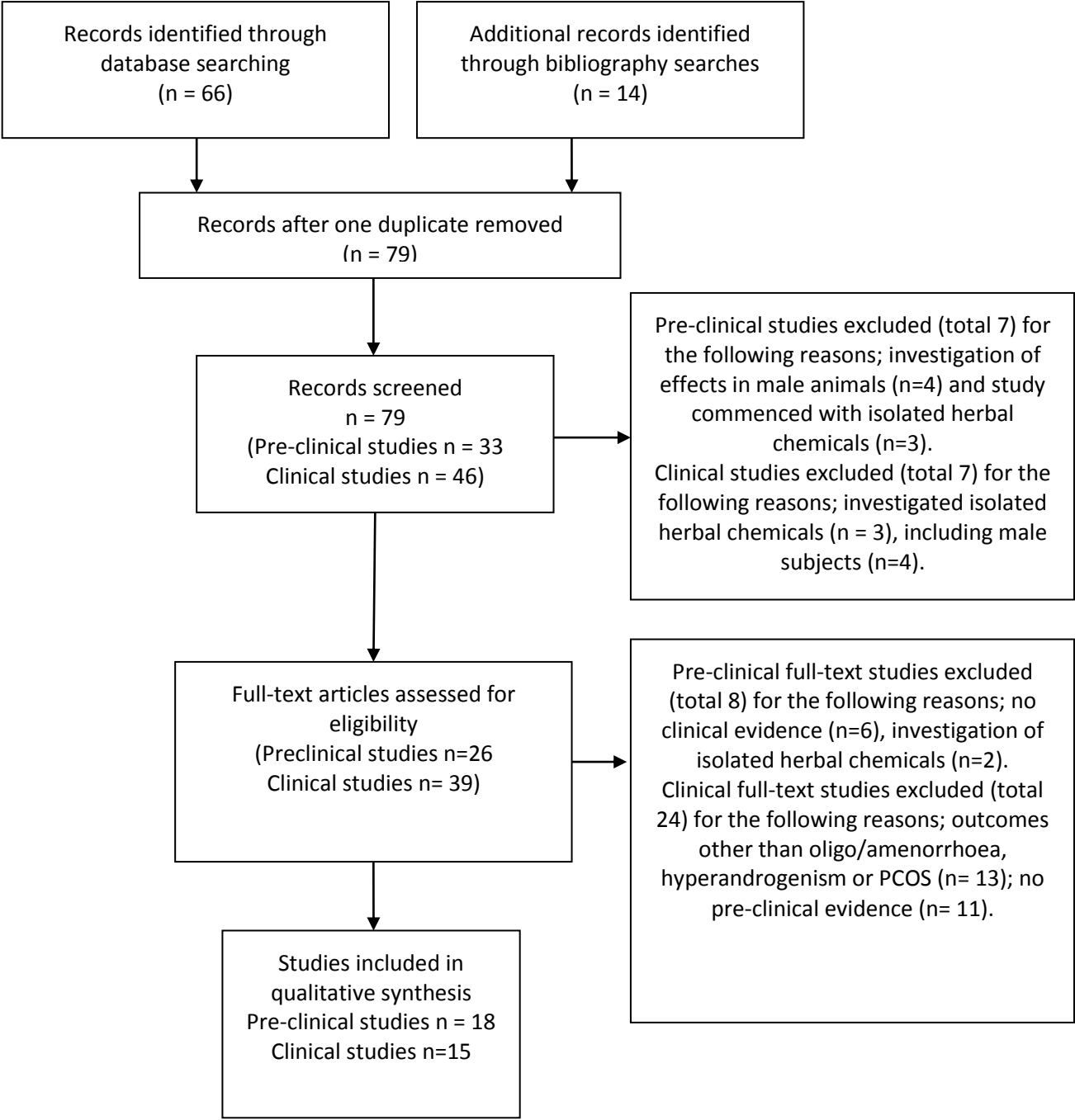

Supplement: Supplementary file 1 — Authors’ original file for figure 1 [file 12906_2014_2122_MOESM1_ESM.pdf]
